# TSH promotes chemerin/CMKLR1–cAMP/ERK–DIO2 signaling in primary rat ependymal cells *in vitro*

**DOI:** 10.1530/JME-26-0028

**Published:** 2026-06-03

**Authors:** Xin Ning, Doudou Guo, Qing Huang, Cencen Wang, Yating Li, Yanfen Zhou, Xin Li

**Affiliations:** ^1^ Department of Pediatrics, Union Hospital, Tongji Medical College, Huazhong University of Science and Technology, Wuhan, China; ^2^ International Medical College, Chongqing Medical University, Chongqing, China

**Keywords:** chemerin, chemokine-like receptor 1, deiodinase activity, thyroid-stimulating hormone, primary ependymal cells

## Abstract

**Abstract:**

In mammals, chemerin and its receptor CMKLR1 might be downstream effectors in response to a long photoperiod in the ependymal cells lining the third ventricle in rats. Iodothyronine deiodinase 2 (DIO2) is a main product of the involvement of TSH in photoperiodic signaling. Thus, the potential interplay of TSH and chemerin/CMKLR1 signaling might exist during photoperiodic changes. Our study aimed to investigate whether chemerin/CMKLR1 signaling could stimulate DIO2 production and how TSH could affect chemerin/CMKLR1 signaling in primary rat ependymal cells. Colocalization of chemerin with its receptor, CMKLR1, was observed using immunohistochemistry assay. The expression of chemerin and CMKLR1 was identified using real-time PCR and western blot analysis, respectively. DIO2 expression, which involves a dual signaling pathway (an increase in cAMP levels and phosphorylation of ERK1/2), was detected by western blot assay and enzyme-linked immunosorbent assay (ELISA). Our study showed the potential interaction between chemerin and CMKLR1 in primary rat ependymal cells. TSH could upregulate the expression of chemerin and CMKLR1 in chemerin-pretreated primary ependymal cells. *In vitro* treatment of primary ependymal cells with chemerin (10 ng/mL) or TSH (60 mIU/mL) could induce an increase in cAMP levels, ERK1/2 phosphorylation and DIO2 expression. Furthermore, we found that TSH could promote the effect of chemerin/CMKLR1 signaling on DIO2 production through an increase in cAMP levels and the phosphorylation of ERK1/2. We concluded that chemerin/CMKLR1 signaling could enhance DIO2 activity in primary rat ependymal cells *in vitro*, and this effect could be promoted by TSH through cAMP and phosphorylation of ERK1/2.

**Main points:**

- Chemerin/CMKLR1 interaction in primary rat ependymal cells is discussed.
- Chemerin/CMKLR1 signals through cAMP and ERK phosphorylation to induce deiodinase 2 expression in primary ependymal cells.
- TSH promotes the effect of chemerin/CMKLR1 signaling on deiodinase 2 expression.

## Introduction

Chemerin, a small chemotactic protein and adipokine, is encoded by the retinoic acid receptor responder 2 (Rarres 2) gene. It has been identified as a natural ligand of three G protein-coupled receptors: chemokine-like receptor 1 (CMKLR1), G protein-coupled receptor 1, and C–C motif chemokine receptor-like 2. Receptor CMKLR1, also known as ChemR23, is widely expressed in various cells and tissues, such as innate immune cells, adipocytes, smooth muscle cells, and cardiomyocytes, as well as in the reproductive system, such as Leydig cells. As the main receptor with high affinity for chemerin, CMKLR1 transduction signaling participates in the pathogenesis of inflammation (1, 2, 3), adipocyte metabolism (4, 5), cardiovascular disorders (6), and reproductive function (7). As a Gi/o protein-coupled receptor, CMKLR1 signals through phosphorylation of extracellular signal-regulated kinase 1/2 (ERK1/2); suppression of cyclic adenosine monophosphate (cAMP) levels; and activation of protein kinase B, NF-κB, and mitogen-activated protein kinase pathways (8, 9, 10, 11). These studies indicate that chemerin and its receptors could exert different biological actions depending on the cell type and internalization signaling.

The ependymal cell is a simple ciliated epithelium lining the ventricular surfaces of the brain and the central canal of the spinal cord. The most common function is thought to maintain the patency of the ventricular system and distribute local metabolites, signaling molecules, and extracellular debris (12, 13). The function of ependymal cells has cell-specific and species-specific variation. The ependymal layer lining the third ventricular of the hypothalamus, consisting of ependymal cells and tanycytes, is functionally unique, where thyroid stimulating hormone (TSH) could bind to TSH receptors and control the expression of type 2 deiodinase (DIO2) in response to photoperiodic changes. TSH transduction signaling in cultured ependymal cells involves an increase in cAMP levels and phosphorylation of ERK1/2 (14, 15). Intracerebroventricular administration of TSH elicits a robust increase in DIO2 expression in the ependymal cells (16, 17). This evidence shows the effect of TSH signaling in ependymal cells on the regulation of DIO2 activity.

In a long photoperiod, the expression of chemerin and CMKLR1 receptor was upregulated in ependymal cell layer and considered as downstream effectors in response to photoperiodic signaling (18, 19). Thus, the potential interplay of TSH and chemerin/CMKLR1 signaling might exist during photoperiodic changes. However, the role of chemerin/CMKLR1 signaling in ependymal cells has not been explored, and it is still unclear whether the potential interaction of TSH and chemerin/CMKLR1 signaling could exist in primary rat ependymal cells *in vitro*.

To elucidate the effect of chemerin/CMKLR1 signaling and their interaction with TSH, we stimulated primary rat ependymal cells with chemerin and TSH *in vitro*, aiming to identify the potential interaction between chemerin and CMKLR1 receptor and to investigate the effect of chemerin/CMKLR1 signaling on DIO2 activity in primary rat ependymal cells.

## Materials and methods

### Primary cell culture

Primary rat ependymal cells were obtained from Cellverse Co., Ltd (RAT-iCell-n021, China). Primary rat ependymal cells were cultured in ependymocyte growth medium supplemented with SingleQuots™ and 1% penicillin–streptomycin (iCell-n021-002r,iCell, China). Cultures were incubated in a humid atmosphere of 5% CO_2_:95% air at 37°C. Viability was confirmed with positive vimentin staining as measured by immunofluorescence microscopy (Supplementary File 1 (see section on Supplementary materials given at the end of the article)). Primary ependymal cells (PECs) were plated in 96-well plates and starved for 6 h in Dulbecco’s modified Eagle’s medium/nutrient mixture F12 without phenol red, supplemented with 0.15% insulin and 0.3% putrescine. A cell suspension was prepared at a density of 2 × 10^5^ cells/mL, and 100 μL of this suspension were added to each well. The slides were placed in 12-well plates, and PECs were seeded in preparation for immunofluorescent staining after drug treatment.

### Primary ependymal cell treatment

Primary ependymal cells were treated with bovine TSH (BSZH Scientific, China) at 60 mIU/mL for 36 h, chemerin (TargetMol Chemicals, USA) at 10 ng/mL for 48 h, and dimethyl sulfoxide (DMSO) only as a control. Chemerin was dissolved in 10% DMSO. Drug treatment was given as follows: i) chemerin + TSH group: PECs were pretreated with chemerin for 12 h, followed by co-treatment with TSH for an additional 36 h; ii) chemerin group: PECs were treated with chemerin for 48 h; and iii) TSH group: PECs were pretreated with DMSO for 12 h, followed by co-treatment with TSH for an additional 36 h. DMSO was treated for 48 h as a control. The concentration of DMSO in each group was controlled at 0.1% DMSO.

### Lentivirus small interfering RNA (siRNA) interference

The mouse CMKLR1 mRNA sequence (GenBank: NM_001359060.1) was analyzed using the strand analysis algorithm (https://www.sigmaaldrich.cn/CN/zh/semi-configurators/shrna?activeLink=productSearch), which predicted the best siRNA design for the CMKLR1, with >90% prediction degradation efficiency. Lentiviral packaging plasmids were used to generate specific siRNAs targeting CMKLR1. Primary ependymal cells at 80% confluency were transfected with 5 μM CMKLR1-targeting siRNA or scramble control using Lipofectamine RNAiMAX transfection reagent, according to the instructions provided by the manufacturer. Sixteen hours after transfection, the cells were starved for 6 h and then treated with TSH and chemerin as described above. The expression of p-ERK and DIO2 and the concentration of cAMP were identified in primary ependymal cells and siRNA-infected ependymal cells. Two- and three-way analyses of variance (ANOVA) were used for multiple comparisons.

### Western blotting

Total proteins were extracted using RIPA buffer containing 1% protease inhibitor. Phosphatase inhibitors were added when phosphorylated proteins were detected. Protein samples were separated using SDS-PAGE and then transferred onto polyvinylidene difluoride membranes. Membranes were blocked with 5% skimmed milk for 1 h at RT and then incubated overnight at 4°C with the following primary antibodies: rabbit p-ERK (1:1,000; CST, USA, AB_2315112), rabbit anti-ERK (1:3,000; CST, USA, AB_390779), rabbit anti-vimentin (1:5,000; Proteintech, China, AB_2273020), and rabbit anti-DIO2 (1:1,000; Abcam, China, AB_1951738). After washing, the membranes were incubated with the secondary antibody for 30 min. Chemiluminescent signals were developed using an ECL kit and detected using the ChemiDoc XRS gel documentation system. Protein bands were analyzed using ImageLab software, and glyceraldehyde 3-phosphate dehydrogenase was used as an internal control. The procedure was performed in 3 triplicates of three independent experiments.

### Immunocytochemistry

After treatment with TSH and/or chemerin as described above, primary ependymal cells were rinsed in phosphate-buffered saline (PBS) and fixed in 4% paraformaldehyde. Fixed cells were incubated overnight at 4°C with a 1:300 dilution of anti-rabbit chemerin monoclonal antibody (Bs-10410r, Bioss, China, AB_3721218) and 1:50 dilution of anti-mouse CMKLR1 monoclonal antibody (Sc-398769, Santa Cruz, USA, AB_3721158) after washing with PBS for three times. The following day, the cells were washed with PBS and incubated with the secondary antibody at 37°C for 40 min. After three PBS washes, the nuclei were stained with 4′,6-diamidino-2-phenylindole (Sigma, USA). Slides were mounted using tri-ethylenediamine (Aladdin, China). The images were captured under 630× magnification using a fluorescence imaging microscope (Olympus, Japan).

### Real-time PCR

Using TRIzol reagent (ELK Biotechnology, China), total RNA was obtained from cells. One microgram of total RNA was reverse transcribed into cDNA using EntiLink™ 1st Strand cDNA Synthesis Super Mix (EQ031, ELK Biotechnology, China). Then, cDNA obtained by reverse transcription and gene-specific primers and a SYBR Green dye kit (EQ031, ELK Biotechnology, China) were used for amplification. Table 1 shows the forward and reverse primers used for the polymerase chain reaction. GAPDH was used as the endogenous control. The amount of mRNA relative to the endogenous control was calculated by the 2^−^^ΔΔ^^Ct^ method.

**Table 1:** Primers used for quantitative reverse transcriptase PCR.

Target gene	Sequence (5′–3′)	
	Forward	Reverse
GAPDH	GCC​AAG​GTC​ATC​CAT​GAC​AAC	GTG​GAT​GCA​GGG​ATG​ATG​TTC
Rarres 2	GAG​GGA​CTG​GAA​GAA​ACC​CG	CCT​TGG​AGA​AGG​CGA​ACT​GT

### cAMP assay

After the cells reached confluence in the original flask, they were plated at a density of 100,000 cells per well in a 24-well plate. After a 48 h recovery period and 16 h overnight serum deprivation, the cells were immediately stimulated with TSH and/or chemerin. DMSO was used as a control. The medium was then removed for the cAMP assay. Protein concentration was determined using the BCA protein assay (AS1086, ASPEN), and extracellular cAMP levels were measured using colorimetric enzyme-linked immunosorbent assay (ELK8116; ELK Biotechnology, China). The optical density was measured using a plate reader at 450 nm. Adenylate cyclase activation assays were performed in 3 triplicates of three independent experiments.

### Statistical analysis

All data were collected from three independent experiments and expressed as mean ± standard error of mean unless otherwise stated in the figure legends. A two- or three-way analysis of variance (ANOVA) was used for multiple comparisons. Tukey’s post hoc test was used following significant ANOVA results. A *P* ≤ 0.05 was considered to indicate a significant difference in mean values between groups.

## Results

### TSH promotes chemerin/CMKLR1 expression in primary rat ependymal cells *in vitro*

**Figure 1:**
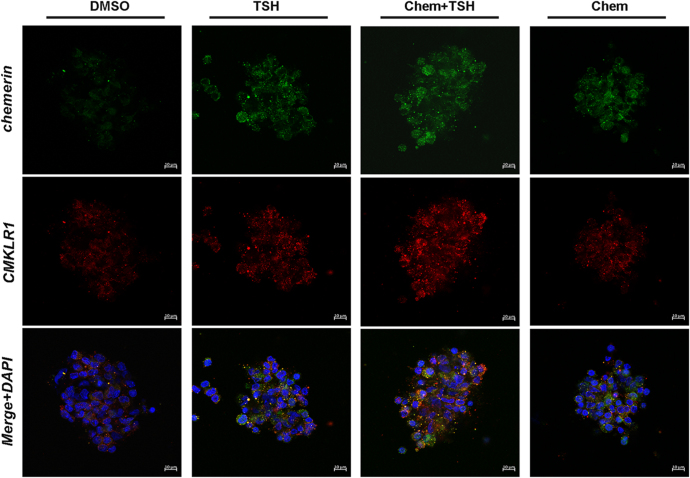
Immunofluorescent staining showing the colocalization of chemerin and its receptor CMKLR1 in primary rat ependymal cells. Primary ependymal cells were treated with chemerin and/or TSH. DMSO was used as a control. (A, B, C, D) The increased expression of chemerin (green) and CMKLR1 (red) was identified using immunofluorescence staining in the chemerin + TSH group, compared to the chemerin group or the TSH group. Nuclei stained with DAPI (blue). Images were taken under 630× magnification. Scale bar: 10 μm.

**Figure 2:**
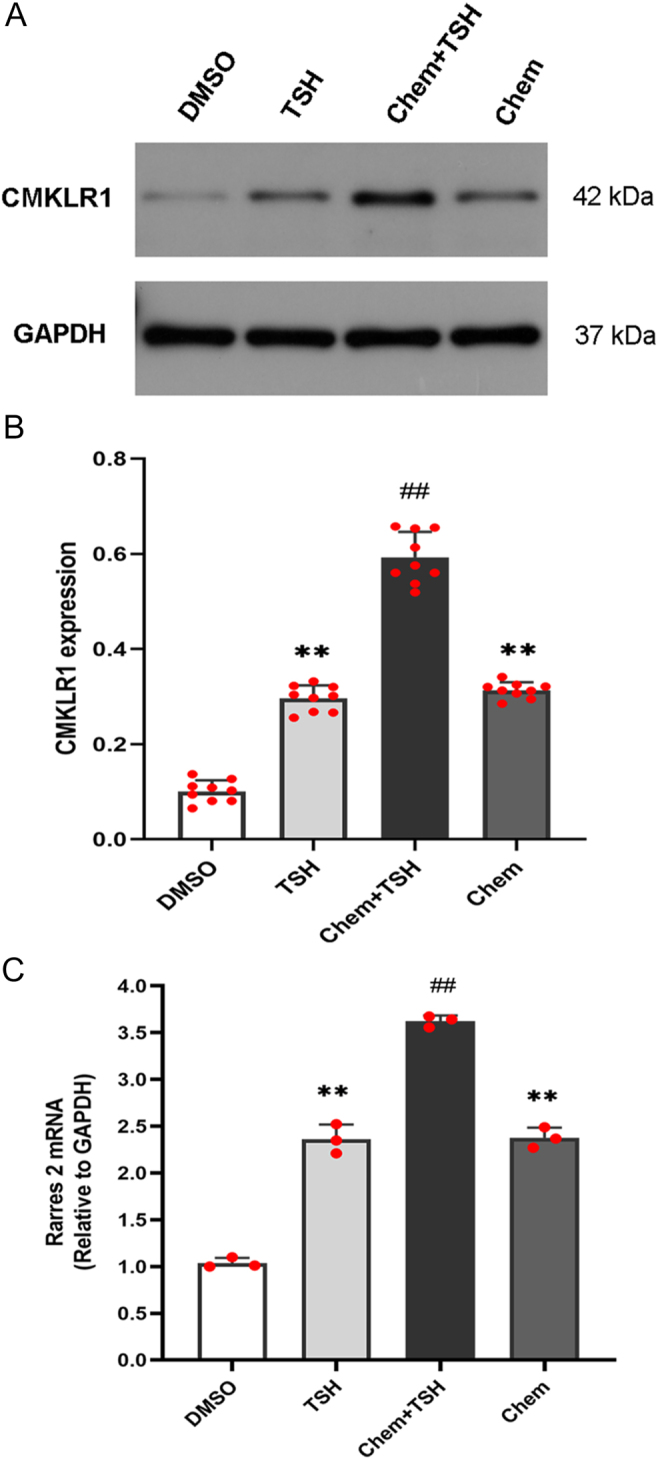
TSH promotes the expression of Rarres 2 mRNA and CMKLR1 receptors in chemerin-pretreated primary ependymal cells. Primary ependymal cells were treated with chemerin and/or TSH. DMSO was used as a control. (A and B) Western blot assay confirmed the upregulation of CMKLR1 receptor in the chemerin + TSH group; (C) Rarres 2 mRNA expression was significantly increased in the chemerin + TSH group, compared to the TSH or chemerin group. *n* = 3 independent experiments. Compared to the chemerin group or the TSH group, ^##^*P* < 0.01; compared to the DMSO group, **P < 0.01. A full color version of this figure is available at https://doi.org/10.1530/JME-26-0028.

To explore the role of TSH in regulating chemerin/CMKLR1 expression in primary ependymal cells, we cultured PECs with bovine TSH and/or chemerin. Immunofluorescence staining showed the colocalization of chemerin and CMKLR1 on ependymal cell membrane, and an increase in the expression of Rarres 2 and CMKLR1 receptor in the chemerin + TSH group compared to that in the TSH group or the chemerin group (Figs 1 and 2). Furthermore, the expression of Rarres 2 mRNA and CMKLR1 receptor was significantly upregulated in the chemerin + TSH group compared to the TSH group or the chemerin group (Fig. 2A, B, C). We also found that CMKLR1 expression in the TSH group was comparable to that in the chemerin group (Fig. 2A, B, C). The result indicates that both chemerin and TSH are capable of promoting CMKLR1 expression in primary ependymal cells.

### TSH promotes DIO2 expression by chemerin signaling through the cAMP/ERK pathway in PECs

**Figure 3:**
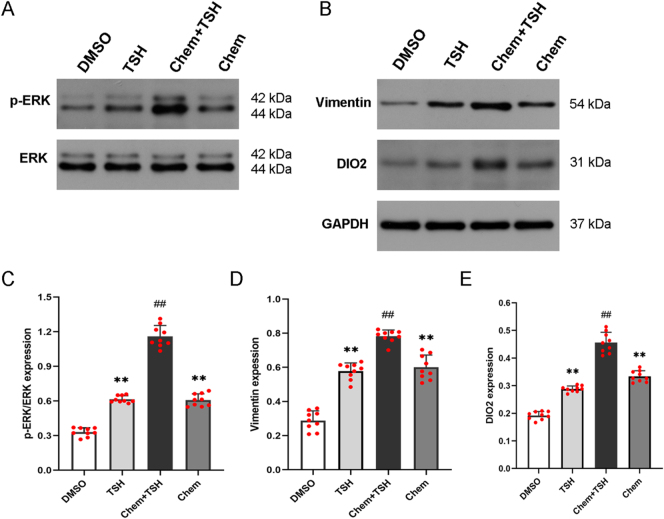
TSH promotes the effect of chemerin on the expression of p-ERK and DIO2 in primary ependymal cells. Primary ependymal cells were treated with chemerin and/or TSH. DMSO was used as a control. (A, B, C, D, E) Western blot analysis showed the upregulation of p-ERK, vimentin, and DIO2 expression in the chemerin + TSH group, compared to the chemerin or TSH group. GAPDH as an internal control; *n* = 3 independent experiments. Compared to the chemerin group or the TSH group, ^##^*P* < 0.01; compared to the DMSO group, **P < 0.01. A full color version of this figure is available at https://doi.org/10.1530/JME-26-0028.

**Figure 4:**
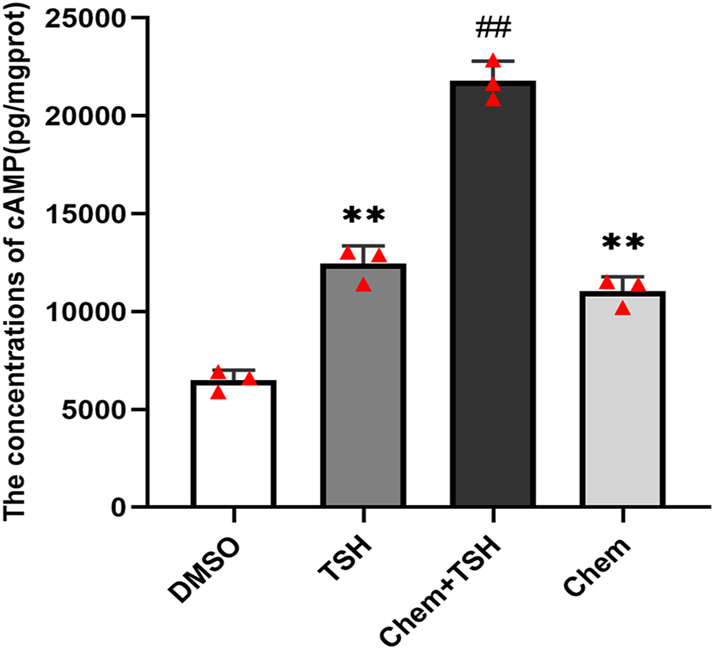
TSH increased the concentration of cAMP by chemerin in primary ependymal cells. ELISA showed the increased concentration of cAMP in the chemerin + TSH group, compared to the chemerin or TSH group. *n* = 3 independent experiments. Compared to the chemerin group or the TSH group, ^##^*P* < 0.01; compared to the DMSO group, **P < 0.01. A full color version of this figure is available at https://doi.org/10.1530/JME-26-0028.

To explore the role of chemerin and TSH in DIO2 expression in primary ependymal cells, total protein was extracted from western blot analysis. The results showed that, compared to the TSH group or the chemerin group, the phosphorylation level of ERK (p-ERK) and the expression of vimentin and DIO2 were significantly increased in the chemerin + TSH group (Fig. 3A, B, C, D, E). The concentration of cAMP in the chemerin + TSH group was higher than that in the TSH group or in the chemerin group (Fig. 4). We also discovered that chemerin could activate the downstream signaling, cAMP and ERK phosphorylation, in PECs. These results suggest that both TSH and chemerin could transduce through cAMP/ERK signaling to induce DIO2 expression, and TSH could further promote the effect of chemerin on DIO2 expression in PECs.

### CMKLR1 knockdown diminished DIO2 expression by chemerin and TSH in PECs

**Figure 5:**
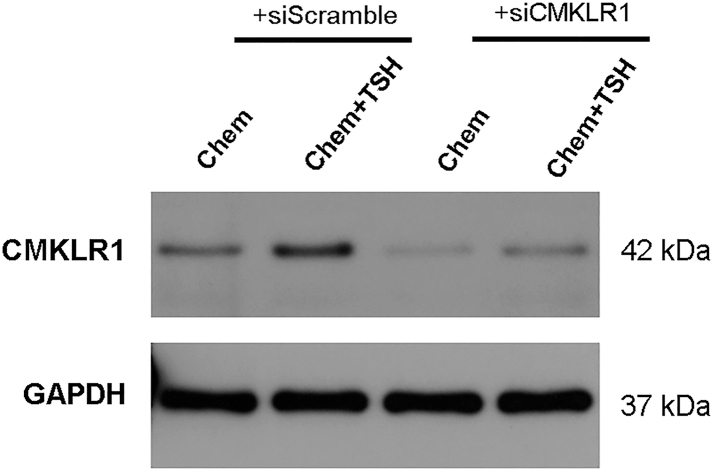
Knockdown of CMKLR1 in primary ependymal cells. Primary ependymal cells were transfected with siRNA targeting CMKLR1 (siCMKLR1) or a control siRNA (siScramble). Twenty-four hours after transfection, primary ependymal cells were treated with chemerin and TSH. Western blot assay confirmed CMKLR1 knockdown in primary ependymal cells.

**Figure 6:**
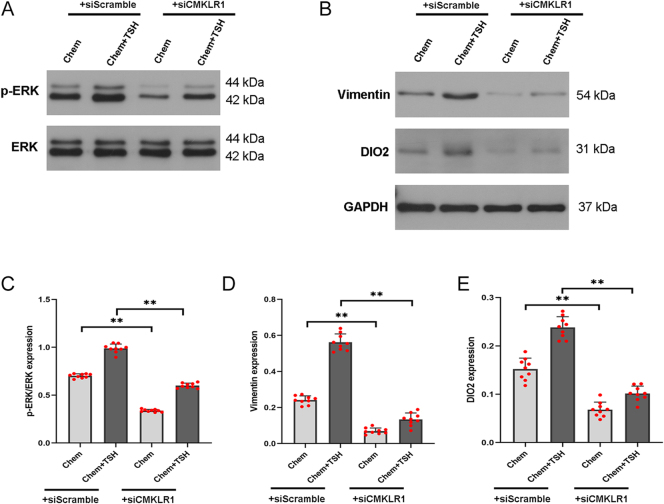
Expression of p-ERK and DIO2 by chemerin and TSH in primary ependymal cells transfected with siRNA against CMKLR1 (siCMKLR1) or a control siRNA (siScramble). (A, B, C, D, E) Western blot analysis showed that the expression of p-ERK, vimentin, and DIO2 was significantly downregulated in chemerin-treated and chemerin + TSH-treated ependymal cells transfected with siCMKLR1, compared to their siScramble controls. GAPDH as an internal control; *n* = 3 independent experiments. Compared to a control siScramble group, **P < 0.01. A full color version of this figure is available at https://doi.org/10.1530/JME-26-0028.

**Figure 7:**
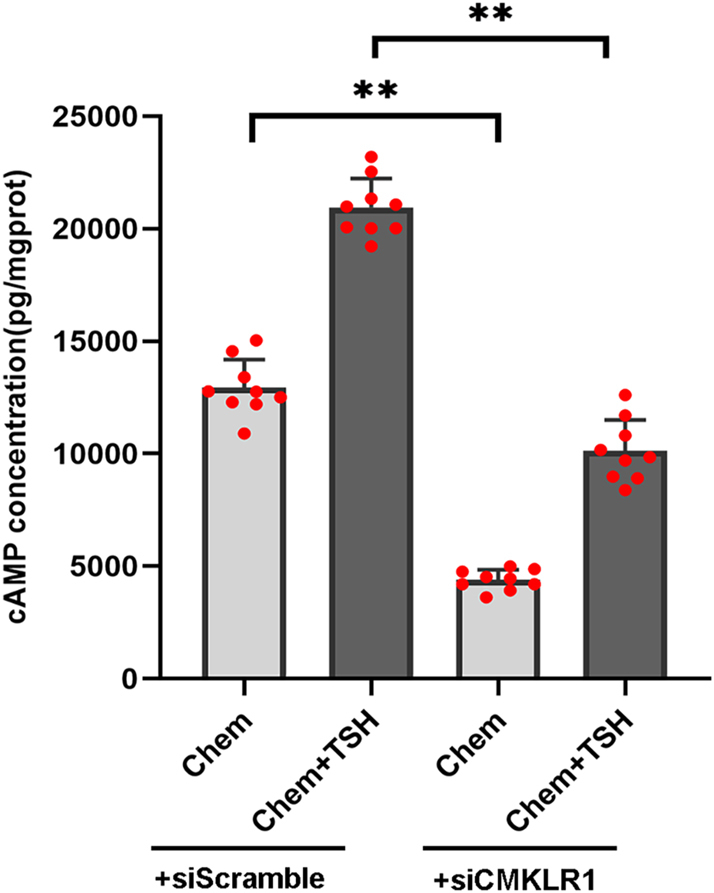
Regulation of cAMP levels by chemerin and TSH in primary ependymal cells transfected with siRNA against CMKLR1 (siCMKLR1) or a control siRNA (siScramble). ELISA showed the decreased concentration of cAMP in chemerin-treated and chemerin + TSH-treated ependymal cells transfected with siCMKLR1, compared to their siScramble control. *n* = 3 independent experiments. Compared to a control siScramble group, **P < 0.01. A full color version of this figure is available at https://doi.org/10.1530/JME-26-0028.

PECs were transfected with siRNA targeting *CMKLR1* (siCMKLR1) to knockdown the CMKLR1 receptor. Twenty-four hours after transfection, the ependymal cells were stimulated with TSH and chemerin. Western blot analysis confirmed CMKLR1 knockdown (Fig. 5). To further investigate the role of CMKLR1 in DIO2 expression in primary ependymal cells, we compared the siCMKLR1 and siScramble control groups. We found that the increased expression of p-ERK, vimentin, and DIO2, as well as the higher level of cAMP concentration by chemerin alone or co-treatment of chemerin and TSH were significantly blocked in the siCMKLR1 group (Figs 6 and 7). CMKLR1 knockdown blocked the activation of the cAMP/ERK pathway and DIO2 expression in both the chemerin group and the chemerin + TSH group. Together, these data suggest that TSH could promote DIO2 expression that chemerin/CMKLR1 signaling could induce through an increase in cAMP level and ERK phosphorylation.

## Discussion

In this study, we demonstrated that TSH could promote the expression of Rarres 2 mRNA and CMKLR1 receptor and synergized with chemerin to enhance its potential interaction with CMKLR1 receptor in primary rat ependymal cells *in vitro*. In addition, chemerin/CMKLR1 could also promote DIO2 expression through cAMP and the phosphorylation of ERK1/2 signaling. Furthermore, TSH could stimulate chemerin/CMKLR1 signaling to increase the transcriptional regulation of DIO2 expression in PECs.

In ependymal layers, chemerin is downstream effector of long photoperiod and retinoic acid (RA) signaling. The integration of the photoperiodic signal involves altered thyroid hormone (TH) driven by TSH. To clarify the *in vitro* effect of TSH on chemerin/CMKLR1 expression, we confirmed for the first time that TSH could stimulate the expression of Rarres 2 and CMKLR1, as indicated by Helfer *et al.* (18). However, it is still unclear how chemerin binds to its receptor CMKLR1 and activates its downstream signaling in ependymal cells.

In many animal species, such as sheep, quail, rat, and hamster, TSH signals through dual signal transduction pathways, Gαs-mediated cAMP production and Gαq-mediated ERK1/2 phosphorylation, to induce DIO2 expression in ependymal layers of the hypothalamus where TSH receptors are expressed (14, 15, 20, 21, 22). In F334 rats, CMKLR1 are expressed in ependymal cells and tanycytes of the hypothalamus *in vivo*. In 57BL/6 mice, chemerin is restricted to ependymal cells, indicating the potential role of chemerin/CMKLR1 in ependymal cells (23). Our data showed that chemerin, like TSH, has the biological capacity for DIO2 expression through an increase in cAMP level and the phosphorylation of ERK in PECs. Furthermore, TSH could promote the effect of chemerin/CMKLR1 signaling on DIO2 expression in primary ependymal cells.

In previous studies, the binding of chemerin to CMKLR1 can trigger multiple signaling cascades. It includes activation of the PIK3 pathway, phosphorylation of ERK1/2, calcium mobilization, and suppression of cAMP accumulation. CMKLR1 interacts with chemerin and displays strong activation and transduction of ERK1/2 signaling in different cells and tissues, such as CHO-K1 cells (8), mouse embryonic fibroblasts (8), preadipocytes (24), porcine pituitary (25), vascular smooth muscle cells (26, 27), and cardiomyocytes (28). Our study indicated that chemerin/CMKLR1 signaling regulates the activation of ERK1/2 in primary ependymal cells, which is consistent with these previous studies. Notably, in CHO-K1 cells, chemerin/CMKLR1 resulted in the inhibition of cAMP (10). Conversely, cAMP levels were significantly increased in primary ependymal cells under the stimulation of chemerin and TSH. This inconsistency may be attributed to the multifaceted role of the chemerin/CMKLR1 axis, which participates in a variety of biological processes through specific transduction pathways in different cell types. First, chemerin-mediated CMKLR1 activation could phosphorylate the intracellular domains of CMKLR1 terminating the signaling pathways by desensitization or internalization (3, 8). Second, CMKLR1-mediated function presents distinct features depending on the activation of chemerin-derived peptides (C9 peptides, C13 peptides, C15 peptides, and C20 peptides) (29). Our data showed the colocalization of chemerin and CMKLR1, indicating their potential interaction. However, more investigations should focus on ligand-binding assay and structural analysis for chemerin binding to CMKLR1 receptor.

We also found that the expression of CMKLR1 was significantly increased in ependymal cultures under the stimulation of chemerin and TSH. The enhancement of the CMKLR1 receptor was also observed in macrophages, where chemerin activated CMKLR1/p-AKT/CEBP signal (30). These previous studies might explain that CMKLR1 expression is significantly upregulated both on the level of TSH treatment and in response to chemerin.

In conclusion, our study is the first to show the role of TSH in the expression of chemerin and CMKLR1 in primary rat ependymal cells. Furthermore, TSH could interact with chemerin/CMKLR1 signaling to further increase DIO2 expression in primary ependymal cells. However, many questions remain regarding the *in vivo* effect of TSH and chemerin on primary ependymal cells. The connection between the intracellular transduction cascade of the chemerin/CMKLR1 axis and TSH signaling will be the focus of future studies.

## Supplementary materials



## Declaration of interest

The authors declare that the research was conducted in the absence of any commercial or financial relationships that could be construed as potential conflicts of interest.

## Funding

This study was supported by the Advanced Foundation Project funded by Wuhan Municipal Administration Bureau of Science and Technology (No.: 2020020601012223).

## Author contribution statement

XN wrote and revised the main manuscript text; DDG, QH, CCW, and YTL prepared Figs 1, 2, 3, 4. YFZ prepared Figs 5 and 6. XL conceptualized and reviewed the manuscript. All authors reviewed the manuscript.

## Data availability

All data generated or analyzed during this study are included in this article. The datasets used and/or analyzed during the current study are available from the corresponding author on reasonable request.
