# Supplementary material for: TSH promotes chemerin/CMKLR1–cAMP/ERK–DIO2 signaling in primary rat ependymal cells in vitro
Source: J Mol Endocrinol. 2026 Jun 3;76(4):e260028. doi: 10.1530/JME-26-0028 (PMC13239433; doi:10.1530/JME-26-0028)
Supplement: Supplementary file 1 [file supplementary_materials.pdf]

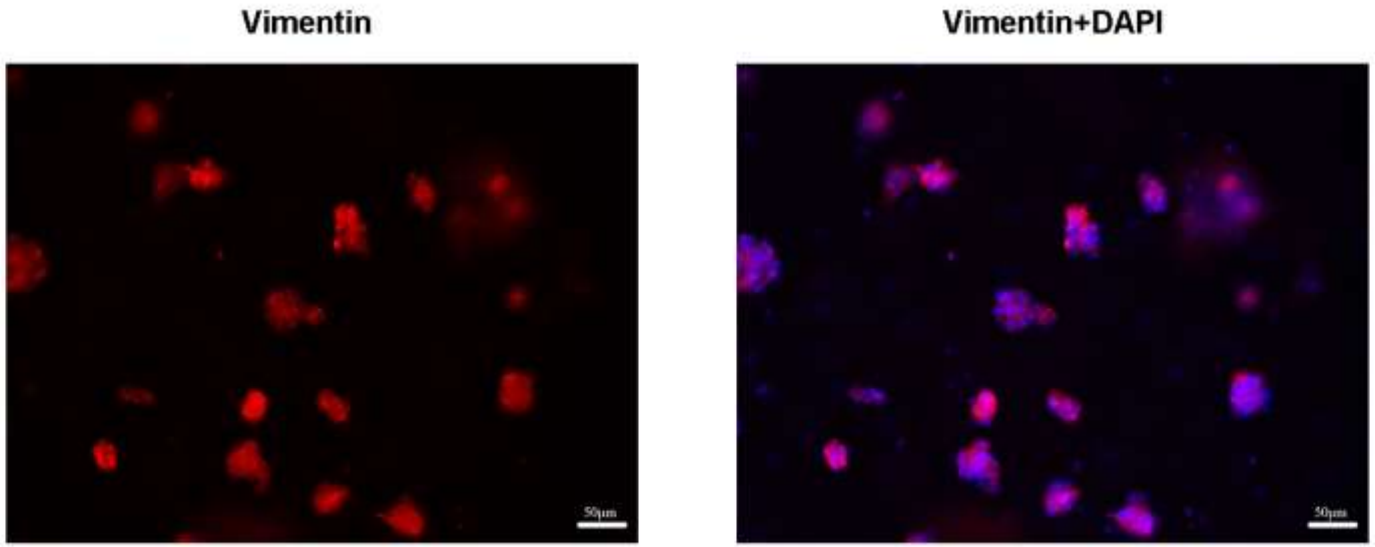


**Supplemental Figure 1.** The expression of vimentin in cultured primary rat ependymal cells. The immunofluorescent staining showed the expression of the intermediate filament protein vimentin(red). Nuclei stained with DAPI (blue). Scale bar:50μm.
